# Optimizing Medication Safety with Oral Antitumor Therapy: A Methodological Approach for the Real-World Implementation of the AMBORA Competence and Consultation Center

**DOI:** 10.3390/healthcare11111640

**Published:** 2023-06-03

**Authors:** Lisa Cuba, Katja Schlichtig, Pauline Dürr, Elisabeth C. Inwald, Martin F. Fromm, Frank Dörje

**Affiliations:** 1 Pharmacy Department, Erlangen University Hospital, 91054 Erlangen, Germany; lisa.cuba@uk-erlangen.de (L.C.); pauline.duerr@uk-erlangen.de (P.D.); 2 Institute of Experimental and Clinical Pharmacology and Toxicology, Friedrich-Alexander-Universität Erlangen-Nürnberg, 91054 Erlangen, Germany; katja.schlichtig@fau.de (K.S.); martin.fromm@fau.de (M.F.F.); 3 Comprehensive Cancer Center Erlangen-EMN, Erlangen University Hospital, 91054 Erlangen, Germany; 4 Department of Gynecology and Obstetrics, University Medical Center Regensburg, 93053 Regensburg, Germany; elisabeth.inwald@ukr.de

**Keywords:** CFIR, implementation science, intensified pharmacological/pharmaceutical care, oncology, RE-AIM, stakeholder interviews

## Abstract

Generating evidence for the efficacy of an intervention is not sufficient to guarantee its implementation in real-world settings. The randomized AMBORA trial (Medication Safety with Oral Antitumor Therapy) demonstrated that an intensified clinical pharmacological/pharmaceutical care program has substantial benefits for patients, treatment teams, and the healthcare system. Thus, we are now investigating its implementation into routine care within the AMBORA Competence and Consultation Center (AMBORA Center). We perform a multicenter type III hybrid trial following the RE-AIM framework to assess the clinical effectiveness of this care program under real-world conditions, while evaluating the implementation outcomes. Semi-structured stakeholder interviews based on the Consolidated Framework for Implementation Research (CFIR) have been conducted to identify barriers and facilitators. So far, 332 patients treated with oral antitumor drugs have been referred to the AMBORA Center by 66 physicians from 13 independent clinical units. In 20 stakeholder interviews (e.g., with clinic directors), 30% (6/20) of the interviewees anticipated possible barriers which may partly hinder sustainable implementation (e.g., unavailable consultation rooms). Furthermore, important facilitators (e.g., operational processes) were identified. This methodological description adds knowledge on how to structure a hybrid effectiveness–implementation trial and proposes multilevel implementation strategies to improve the medication safety of oral antitumor therapy.

## 1. Introduction

Generating evidence for the efficacy of an intervention is not sufficient to guarantee its transfer into routine care [1,2]. Not even 50% of clinical innovations make it into real-world settings, and for those overcoming the gap between research and practice, an excessively large latency of 17 to 20 years is estimated by several studies [3,4,5,6]. Eighty-five percent of medical research funds do not have a long-term impact on public healthcare systems [7], resulting in an evidence–practice gap which deprives patients of new clinical knowledge [8].

Oral antitumor therapeutics (OAT) represent a rapidly growing type of cancer treatment across multiple tumor entities, ranging from solid tumors to hemato-oncological diseases [9]. More than 100 substances are approved as orally available anticancer treatments in Germany as of April 2023. Advantages such as convenience and independent drug application without the need for intravenous access, as well as increased flexibility, make OAT attractive for both the patients and treatment teams. However, this type of treatment is also associated with several major challenges, e.g., complex intake regimens, difficult handling instructions, and the risk of drug–drug or drug–food interactions, which can either threaten desired therapeutic outcomes or increase the risk of potential adverse events [10,11,12].

To meet these challenges regarding treatment with OAT, an intensified clinical pharmacological/pharmaceutical care program was developed and successfully evaluated in the randomized, multicenter AMBORA trial (Medication Safety with Oral Antitumor Therapy, DRKS00013271) [13]. In brief, patients with a newly started OAT regimen were randomized to either receive standard clinical care (control group) or the intensified AMBORA care program, which included four specified consultation sessions at predefined time points within 12 weeks (intervention group) [13]. No tumor entities or stages were excluded, covering a broad range of patients [13]. The number of drug-related problems (unresolved medication errors and side effects) was significantly reduced by 34% and patient satisfaction increased. The probability of reaching a combined endpoint consisting of four highly relevant clinical outcomes (severe side effects, therapy discontinuation, hospitalization, or death) was significantly reduced by 52% in the intervention group [13]. Additionally, two in-depth analyses of medication errors in the overall AMBORA population [14] and the high-risk uro-oncological population [15] indicated the need for an improvement of medication safety. In conclusion, patients and treatment teams should be addressed to optimize medication safety with OAT [13,14]. Moreover, the AMBORA care program had positive effects on the healthcare system by reducing the cost via cutting the amount of wasted, expensive oral antitumor medications due to treatment discontinuation [16] or reduced hospitalization rates [17].

The very promising results of the AMBORA trial [13] provide striking evidence for the need to improve the medication safety of OAT with an additional intensified clinical pharmacological/pharmaceutical care program. To address the need for enhanced transfer of innovations, the field of implementation science has evolved [18] and is defined as the study of strategies used to bring evidence-based practices into real-world clinical care [2,19]. Implementation research makes use of multiple methods to evaluate and accelerate the uptake of innovations in order to ultimately improve the standard of care. Despite the growing interest in the field of implementation science, few studies systematically report their methodological approaches [2]. Limited data focusing on implementation science in the context of OAT are available. To close the apparent gap between research and practice, we now investigate the implementation of the evidence-based AMBORA care program [13] into routine clinical care as a key component of the AMBORA Competence and Consultation Center (AMBORA Center), using an implementation science approach.

## 2. Materials and Methods

### 2.1. Setting

This ongoing, real-world implementation trial is conducted within the Comprehensive Cancer Center (CCC) Erlangen-EMN. The AMBORA Center has been rolled out to multiple University hospital outpatient clinical units and practices for hematology and oncology that treat various tumor entities and differ in their respective patient volumes, as well as organizational structures. Following a positive vote by the ethics committee of the Friedrich-Alexander-Universität (FAU) Erlangen-Nürnberg, the investigation was registered at the German Clinical Trials Register (DRKS00026272). Patient healthcare data confidentiality is assured by data protection rules. Data are aggregated in a pseudonymized fashion. Written informed consent is obtained from all subjects involved in the additional optional evaluation of patient-reported outcomes (PRO). The present investigation describes the methodological approach and the preliminary results of the implementation of the AMBORA Center to optimize the medication safety of OAT in a real-world setting.

### 2.2. Study Design and Patients

The AMBORA Center emphasizes the evaluation of implementation outcomes, while concurrently assessing clinical effectiveness outcomes. Hence, it can be classified as a non-randomized type III hybrid effectiveness–implementation trial [20]. By identifying barriers and facilitators and gathering information on the utility of different implementation strategies in multiple clinical units, we aim to derive a best practice model of how to foster a successful and sustainable integration of the intensified AMBORA care program into routine clinical practice.

**Figure 1:**
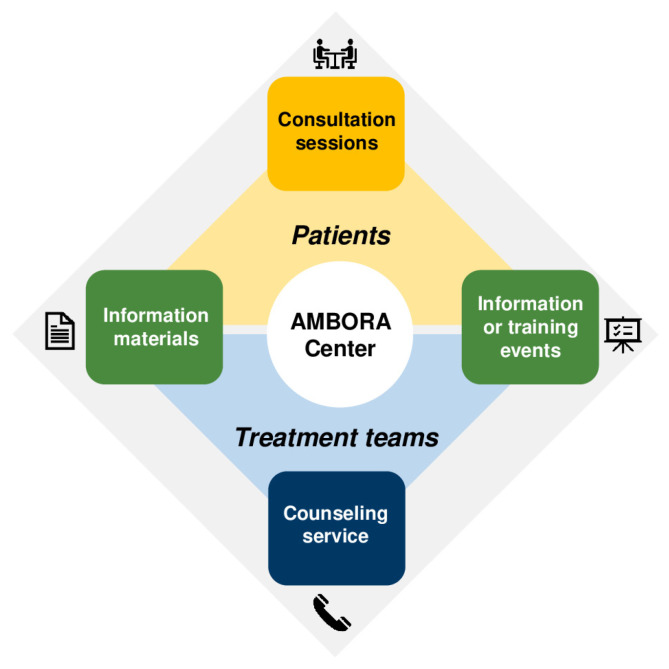
Overview of services provided by the AMBORA Center.

Within the randomized AMBORA trial [13], only patients starting treatment with a new OAT regimen (defined as any OAT approved in Germany since imatinib in 2001) were eligible. Following the transition into real-world clinical practice, we set broader inclusion criteria: all patients treated with any OAT (including longstanding cytostatic drugs, e.g., cyclophosphamide) can be consulted by the AMBORA Center at OAT initiation or any timepoint during an ongoing therapy. Besides structured patient consultation sessions in line with standard operating procedures (SOP) of the AMBORA trial [13] as a key component, the AMBORA Center offers a counseling service for healthcare professionals (e.g., physicians and nurses) and information material (e.g., antitumor drug fact sheets or information brochures about common adverse events), as well as information or training events for both patients and treatment teams (Figure 1).

### 2.3. Implementation Process and Strategies

RE-AIM is applied as our conceptualizing and evaluation framework to structure the implementation process [21]. RE-AIM consists of five dimensions (*Reach*, *Effectiveness*, *Adoption*, *Implementation*, and *Maintenance*) and focuses not only on the individuals intended to benefit from the intervention, but also addresses the surrounding levels (e.g., staff and organizations) [21,22]. While the *Reach*, *Effectiveness*, and *Maintenance* dimensions target the individual level (for example, the number of participating patients, the impact of the intervention on clinical outcomes, and the extent to which an intervention becomes part of routine care), the *Adoption* and *Implementation* dimensions operate at the context level (for examples, the number of physicians who implement the intervention into clinical practice and the perception and delivery of the intervention) [21].

**Figure 2:**
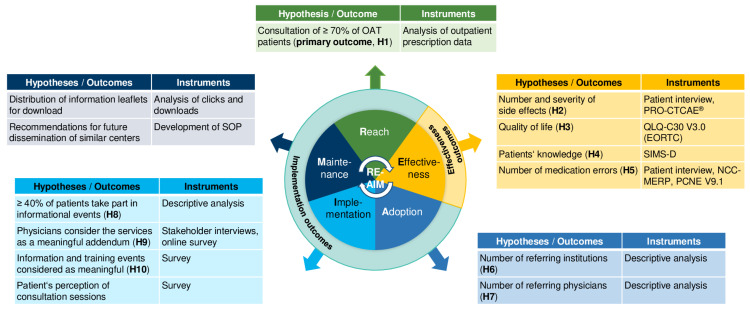
Hypotheses, outcomes, and evaluation instruments assigned to the respective RE-AIM [21] dimensions. Abbreviations: H = hypothesis, NCC-MERP = National Coordinating Council for Medication Error Reporting and Prevention [24], OAT = oral antitumor therapeutics, PCNE V9.1 = Pharmaceutical Care Network Europe [25], PRO-CTCAE^®^ = Patient Reported Outcomes version of the Common Terminology Criteria for Adverse Events [26], QLQ-C30 V3.0 (EORTC) = European Organization for the Research and Treatment of Cancer Quality of Life Questionnaire [27], SIMS-D = Satisfaction with Information about Medicines Scale, German version [28], SOP = standard operating procedures.

We defined ten hypotheses (**H1**–**H10**) prior to the roll-out of the AMBORA Center and assigned them to their respective RE-AIM dimensions (Figure 2). They are further categorized into implementation (**H1**, **H6**–**H10**) and effectiveness (**H2**–**H5**) outcomes. All methodological approaches were developed and discussed within a steering committee (including a patient representative, an expert in the field of implementation and healthcare research, two representatives of the CCC Erlangen-EMN, and the AMBORA Center team) and are further described below. A detailed report according to the Standards for Reporting Implementation Studies (StaRI) guidelines [23] is presented in the Supplementary Materials (Figure S2).

#### 2.3.1. Dimension Reach

**H1** **(primary** **outcome).**  *At least 70% of patients treated with OAT at the CCC Erlangen-EMN are counseled by the AMBORA Center*.

Patient recruitment is regularly assessed and monitored to identify highly or poorly recruiting institutions and the respective barriers and facilitators (e.g., certain organizational structures). We use several strategies to enroll as many patients as possible (e.g., screening of patient lists). Outpatient prescription data will be used to estimate whether the primary endpoint is met.

#### 2.3.2. Dimension Effectiveness

**H2.**  *The number and severity of side effects related to the OAT improve over time*.

**H3.**  *The health-related quality of life of the patients is not reduced over time*.

**H4.**  *The patients’ knowledge about the OAT is improved over time*.

**H5.**  *The number of medication errors related to the OAT is reduced over time*.

As mentioned above, the AMBORA Center offers structured clinical pharmacological/pharmaceutical patient consultation sessions in concordance with the published and validated methods [13], addressing four main topics: detailed education about the OAT, management of side effects, thorough medication reconciliation, and adherence support. Side effects (**H2**) and medication errors (**H5**) will be evaluated in all patients using SOP and data obtained in the structured interviews, and supplemented via medical records (for details on the conducted patient consultations and medication reviews, see Dürr et al. [13]). Medication errors are characterized regarding the severity and cause, using validated tools [24,25]. Patients starting oral antitumor therapeutics (OAT) are eligible for an additional, optional evaluation using validated questionnaires to assess patient-reported outcomes (PRO): the Patient Reported Outcomes—Common Terminology Criteria for Adverse Events (PRO-CTCAE^®^) version 1.0 [26], which measures the symptom burden (H2); the European Organization for Research and Treatment of Cancer (EORTC) QLQ-C30 version 3.0 [27], which evaluates the quality of life (H3); and the Satisfaction with Information about Medicines Scale (SIMS-D [28]), which assesses patients’ knowledge (H4). All PRO instruments are assessed at two different timepoints to evaluate changes over time: SIMS-D at the initial consultation and first follow-up, and PRO-CTCAE and QLQ-C30 twice within the follow-up. To gather a deeper understanding of why patients refuse to complete the additional PRO questionnaires, individual reasons for refusal are surveyed.

#### 2.3.3. Dimension Adoption

**H6.**  *The number of institutions referring to the AMBORA Center is at least as high as in the AMBORA trial [13]*.

**H7.**  *The numbers of physicians referring to the AMBORA Center is at least as high as in the AMBORA trial [13]*.

**Figure 3:**
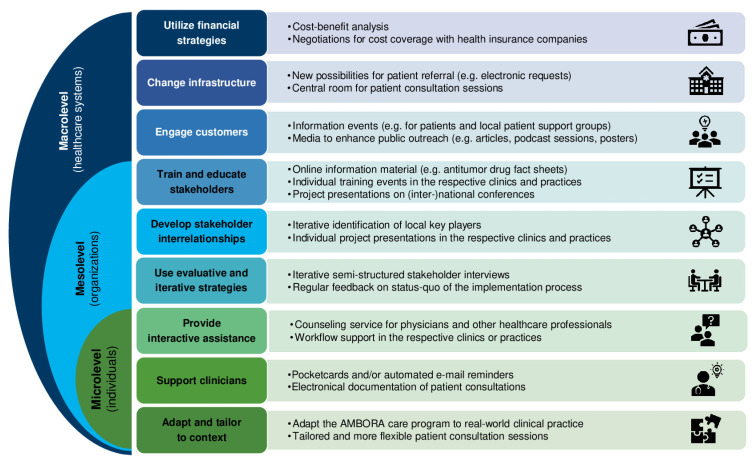
Selection of implementation strategies sorted according to the taxonomy of the ERIC study [29] and their respective levels [30].

The degree of adoption by physicians and the respective clinics and practices is analyzed descriptively. To enhance the outreach of the AMBORA Center within CCC Erlangen-EMN and beyond, diverse implementation strategies are developed, applied, and adjusted. Following the taxonomy of Waltz et al. [29], we assigned our implementation strategies to the corresponding categories (Figure 3). Furthermore, we assorted the respective categories according to Wirtz et al. [30] in order to distinguish between micro-, meso-, and macrolevel strategies used to address individuals, the organization, and the overarching systems (Figure 3).

#### 2.3.4. Dimension Implementation

**H8.**  *At least 40% of patients counseled by the AMBORA Center take part in the informational events for patients no less than once*.

**H9.**  *Physicians consider the services of the AMBORA Center as a meaningful addendum in routine medical care for patients’ health*.

**H10.**  *The offered information events, as well as basic and advanced training sessions are considered meaningful by the majority of participants*.

In addition to descriptive analyses of all stated hypotheses, **H9** is evaluated by an anonymized online survey to determine the healthcare professionals’ (e.g., physicians and nurses) perception of the provided services (e.g., concerning patient consultation sessions, counseling service for healthcare providers, and information leaflets). Gathering the basic data of participating healthcare professionals (e.g., age and working experience of physicians and nurses) will allow us to identify certain characteristics of providers who use or do not use our services. **H10** is additionally evaluated by anonymized surveys after the respective events. Moreover, questionnaires are offered to patients after the clinical pharmacological/pharmaceutical consultation sessions to evaluate the patients’ perception. Since a thorough literature review revealed the lack of appropriate, validated evaluation tools, we use self-developed questionnaires for these purposes.

#### 2.3.5. Semi-Structured Stakeholder Interviews

As another important part of our implementation strategy, we placed a special focus on a qualitative evaluation by conducting semi-structured stakeholder interviews at all participating clinics and practices. We defined stakeholders as clinic directors, leading physicians, or resident practice owners. The interviews were conducted with at least two stakeholders per clinic or practice where possible.

The interviews include questions based on the Consolidated Framework for Implementation Research (CFIR) [31] and are conducted at two timepoints: initial launch of the AMBORA Center (interview #1) and after about 12 months of ongoing patient recruitment (interview #2) in the respective clinic or practice. The CFIR is a determinant framework consisting of 39 (sub-)constructs across five major domains (*intervention characteristics*, *outer setting*, *inner setting*, *characteristics of the individuals involved*, and *process of implementation*) that are considered to influence the implementation outcomes [31].

**Figure 4:**
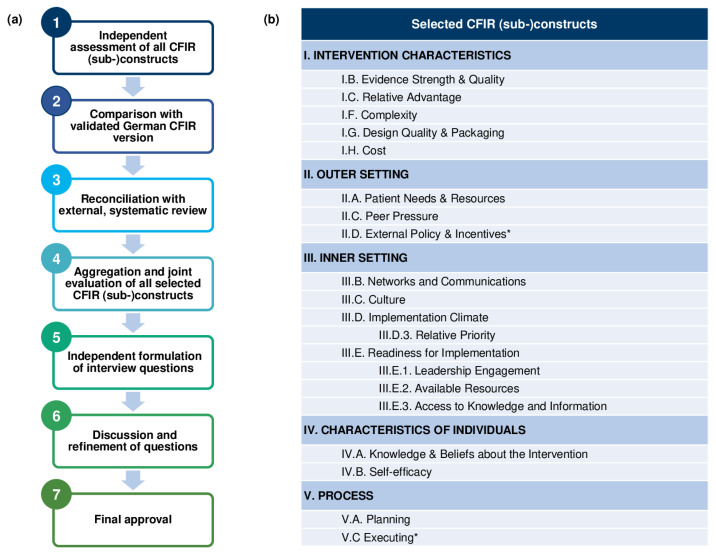
Presentation of (**a**) the multilevel methodological approach for developing semi-structured stakeholder interview questions based on the Consolidated Framework for Implementation Research (CFIR) [31] and (**b**) the selected CFIR (sub-)constructs for the formulation of the interview questions. * Constructs II.D. and V.C. are only taken into account in stakeholder interview #2.

The multilevel methodological approach (e.g., using the validated German CFIR version [32] and a systematic review [33]) for developing our semi-structured interview questions and the selected CFIR (sub-)constructs [31] is displayed in Figure 4. A more detailed methodological description is reported in the Supplementary Materials. Gathering implementation-centered qualitative data by conducting semi-structured stakeholder interviews will allow us to (1) identify barriers and facilitators at an early stage, (2) see trends in the translation into real-world settings, and (3) optimize tailored implementation strategies within the respective clinics and practices.

#### 2.3.6. Dimension Maintenance

We attribute the overarching goal of this type III hybrid effectiveness–implementation trial to the RE-AIM dimension *Maintenance*. For example, all information materials are publicly available for free downloading [34] at a supra-regional level. An analysis of clicks and downloads will be used to identify effective strategies to enhance the outreach. Moreover, we aim to give recommendations for the dissemination and sustainable implementation of similar clinical pharmacological/pharmaceutical care programs at other sites, and plan to perform a cost analysis based on time and personnel requirements.

### 2.4. Statistical Analysis

Descriptive statistics were used to compute patients’ demographic and clinical characteristics and the number of referring institutions/physicians, and to analyze the results of stakeholder interview #1. Microsoft Access^®^ was used for data storage and Microsoft Excel^®^ was used for data preparation and statistical analyses. For the primary endpoint (at least 70% of patients treated with OAT at the CCC Erlangen-EMN are counseled by the AMBORA Center, **H1**), missing data observations do not apply, as outpatient prescription data are available for all participating clinical units. Drug-related problems (e.g., the number of side effects, **H2** and medication errors, **H5**) are assessed at every patient contact and thereby available at least once. As the follow-up contacts are less strict and more variable compared to the AMBORA trial, at least one time point of data collection is sufficient to include patients in the analyses. For patient-reported outcomes (SIMS-D [28], QLQ-C30 [27], and PRO-CTCAE [26]), missing items are handled following the manuals of the corresponding scores. The Institute of Medical Biometry, Informatics and Epidemiology (University Bonn, Germany) provides statistical support for the descriptive analyses of both the effectiveness and implementation outcomes.

## 3. Results

In the following sections, we provide qualitative and quantitative interim data associated with the *Reach* (primary outcome, **H1**), *Adoption* (**H6**–**H7**), and *Implementation* (**H8**–**H10**) dimensions as of 31 January 2023. The hypotheses and outcomes attributed to the *Effectiveness* (**H2**–**H5**) and *Maintenance dimensions* will be reported after data completion.

### 3.1. Dimension Reach

In total, 332 patients treated with 60 different OAT have been referred to the AMBORA Center so far. Overall, the patients were on average 63 (25–88) years old (mean, range) and 57.5% (191/332) were female. Patients took a median number of nine (1–24) drugs (median, range), including prescribed concomitant medication, as well as over-the-counter (OTC) drugs and dietary supplements. Remarkably, a regular consumption of OTC products and grapefruit products was reported by 62.0% (206/332) and 12.0% (40/332) of all patients, respectively. Approximately half of the patients (50.3%, 167/332) were consulted directly at the initiation of the OAT and thereof, 34.7% (58/167) took part in the additional evaluation of patient-reported outcomes (PRO) using validated questionnaires. In total, 65.3% (109/167) of patients with a newly initiated OAT regimen did not participate in the additional PRO evaluation using the three validated questionnaires. Initial consultations were performed using a phone for 10.2% (17/167) of the patients, thus precluding written informed consent. Most frequently, “too complex” was stated as the individual reason for refusal (26.3%, 44/167). Moreover, linguistic or cognitive barriers hindering the completion of the questionnaires were observed in 7.2% (12/167) and 3.6 (6/167) of patients, respectively.

### 3.2. Dimension Adoption

Patients were referred by 13 independent clinical units and practices associated with the CCC Erlangen-EMN (**H6**). We included multiple university hospital outpatient clinics treating various tumor entities (ranging from solid tumors to hemato-oncological diseases). The referring units differ in their respective patient volumes: clinics with a high number of patients (e.g., gynecology, urology, or dermatology) are included, as well as highly specialized outpatient units (e.g., for neuroendocrine tumors) or practices for hematology and oncology. Moreover, we included clinical units with different organizational structures. So far, 66 different physicians have been involved in the recruitment of patients (**H7**), ranging from assistant physicians (interns) to senior physicians or clinic directors. Detailed data on the physicians’ medical specialty and work experience will be gathered by an anonymized online survey (see Figure 2).

### 3.3. Dimension Implementation 

**Figure 5:**
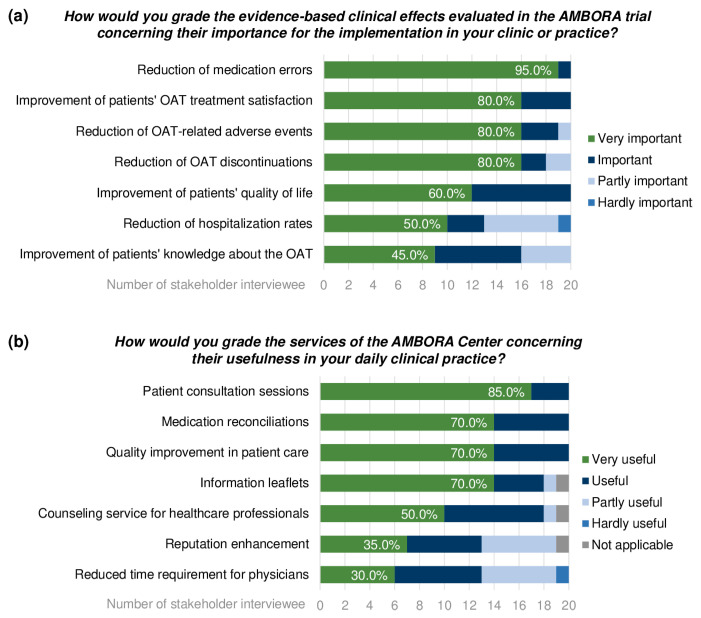
Analysis of the selected questions discussed in semi-structured stakeholder interviews (#1) concerning (**a**) clinical effects evaluated in the AMBORA trial [13] and (**b**) the usefulness of services provided by the AMBORA Center. Abbreviations: OAT = oral antitumor therapeutics.

In total, 20 semi-structured stakeholder interviews (#1) were performed with leading physicians (*n* = 13), clinic directors (*n* = 5), and resident practice owners (*n* = 2) at the launch of the AMBORA Center at all participating clinics and practices (**H9**). The English translation of the stakeholder interview guide, including all proposed questions, is provided in the Supplementary Materials (Figure S1). The interviews lasted on average 32 min (range 20–60 min). During the face-to-face interviews, the stakeholders assessed the evidence-based clinical effects evaluated in the AMBORA trial [13] (Figure 5a) and stated their personal ranking of the offered services of the AMBORA Center concerning their usefulness in the stakeholders’ daily routine (Figure 5b). The reduction in medication errors (95.0%, 19/20) and performance of patient consultation sessions (85.0%, 17/20) was considered to be of most important evidence (Figure 5a) and most useful real-world clinical practice (Figure 5b).

The majority of stakeholders considered the implementation of the AMBORA Center in routine care as definitely organizationally feasible (70.0%, 14/20). However, 30.0% (6/20) of the interviewees anticipated possible barriers partly hindering sustainable implementation and a seamless transition into a real-world setting, including limited access to patient records (60.0%, 12/20), unavailable rooms for adequate clinical pharmacological/pharmaceutical patient consultations in the clinics or practices (45.0%, 9/20), and limited time or personnel resources for patient referral (25.0%, 5/20) as the most common. On the other hand, operational processes (e.g., certain consultation hours; 40.0%, 8/20), newly established infrastructure (e.g., rooms for consultations; 40.0%, 8/20), and communication paths (e.g., automated reminders for patient referral; 30.0%, 6/20) were identified to serve as important facilitators of the implementation process.

## 4. Discussion

In the U.S., performance measures for OAT were first introduced in 2013 (ASCOs Quality Oncology Practice Initiative (QOPI)), but the latest quality analysis revealed a wide variability in the performance across the participating practices [35]. The authors of this recent quality analysis concluded that their findings “highlight the need for the development and implementation of appropriate standards that apply to oral chemotherapy and address the complexities that set it apart from parenteral treatment“ [35]. In particular, a high value has been attributed to clinical pharmacologists/pharmacists within an interprofessional oncologic healthcare team, playing a central role in the improvement of medication safety [36,37]. Similar initiatives including OAT quality measures are still lacking in German healthcare settings.

Here, we present the methodological approach and first results of the real-world implementation of the AMBORA Center, to optimize the medication safety of oral antitumor therapeutics. As a key part, we investigate the implementation of an evidence-based clinical pharmacological/pharmaceutical care program evaluated in the randomized AMBORA trial [13] into routine clinical care. Our investigation is designed as a multicenter type III hybrid trial, and precisely addresses the gap between evidence and practice by blending implementation and effectiveness outcomes.

We use RE-AIM (*Reach*, *Effectiveness*, *Adoption*, *Implementation*, and *Maintenance*) as our conceptualizing framework. After an interim analysis of the *Reach* dimension, we can state that 332 patients treated with 60 different OAT were consulted at the AMBORA Center so far. The patients were referred by 66 physicians from 13 independent clinical units. Hence, we can conclude that hypotheses **H6** and **H7** (*Adoption* dimension) are already met, as we have observed a higher number of referring clinics/practices and physicians in comparison to the AMBORA trial (11 independent university outpatient clinical units and 48 physicians [13]). The hypotheses and outcomes attributed to *Effectiveness* (**H2**–**H5**) and *Maintenance* dimensions will be reported after data completion. Multiple theories, models, and frameworks have been developed to structure and facilitate implementation studies [38,39]. Here, we state our methodological approach using RE-AIM (Figure 1), which has been widely used in healthcare systems [21] (e.g., in the field of oncology investigating the implementation of telehealth [40], in the dissemination of a lung cancer screening education program [41], or in cancer-specific exercise programs [42]). However, RE-AIM does not incorporate contextual factors, or give recommendations to enhance outcomes [43]. To determine why implementation succeeded or failed, the CFIR [31] can be used as a determinant framework to proactively identify barriers or facilitators, to promote *Adoption*, *Implementation*, and *Maintenance.* Therefore, we combine different conceptualizing and determinant frameworks, as described similarly in previous studies [44,45].

We used the CFIR to develop semi-structured stakeholder interview questions. In 20 conducted stakeholder interviews (e.g., with clinic directors) at an early stage of the implementation process, 30% (6/20) of the interviewees anticipated possible barriers that could partly hinder sustainable implementation (e.g., unavailable rooms for consultations). Furthermore, important facilitators (e.g., operational processes) of the implementation process were identified. These results valuably contribute to a sustainable integration of the AMBORA Center. Based on the present formative evaluation, we consider stakeholder interviews a central tool to optimize translation into routine care alongside several other tailored implementation strategies. Systematic implementation science approaches consisting of various interventions and strategies, along with clearly defined outcomes and objectives, are required to deliver high-quality care to patients treated with OAT [46]. Interprofessional interviews similar to our semi-structured stakeholder interviews are often included in these approaches, but data on the practical development of the questions are rare. Verot et al. [47] conducted 20 semi-structured stakeholder interviews at three comprehensive cancer centers in France using the Theoretical Domains Framework. Kinnaer et al. [48] performed 47 semi-structured interviews at four non-academic hospitals in Belgium. Although both studies analyzed the implementation of an interprofessional care program for patients treated with OAT, the studies are difficult to compare with our AMBORA Center because they focused on other healthcare professional groups and lack effectiveness outcomes. A recent single-arm pilot study [49] recruiting 40 hemato-oncological patients treated with OAT assessed the implementation of a model integrating primary and oncology pharmacists by performing interviews, but neither applied systematic frameworks nor included effectiveness outcomes.

Taken together, none of the described studies were designed as hybrid trials blending research on implementation outcomes on the one hand, and the effectiveness of an evidence-based intervention in real-world settings on the other hand. Nevertheless, our methodological approach has some limitations to be aware of. First of all, we systematically selected only 18 of 39 CFIR (sub-)constructs out of all five major CFIR domains to be included in the stakeholder interviews due to time (Figure 4b). Indeed, Damschroder et al. [31] postulated that researchers should select CFIR (sub-)constructs they consider most relevant to their particular study setting, and a systematic review by Kirk et al. [33] encourages reporting on which and how CFIR (sub-)constructs were chosen. We accurately report our selection of CFIR (sub-)constructs in Figure 4a and in the Supplementary Materials. Secondly, the semi-structured, face-to-face interview format itself is associated with bias due to the presence of AMBORA Center team members. Interviews were not audiotaped in order to allow for a time-efficient dialogue and avoid a surveillance atmosphere. Nevertheless, we chose this format because it enables open-minded and tailored discussion. Moreover, the generalizability of our investigation to other settings might be limited due to its conduction within the German healthcare system (e.g., filling of OAT prescriptions for outpatients is performed by any community pharmacy and not by hospital pharmacies). 

Further research regarding the impact on medication safety with oral antitumor therapy in real-world settings and the feasibility in different routine clinical settings is required. As a next step, we plan to derive recommendations (e.g., a best practice model) for the dissemination of similar AMBORA Centers to other sites in Germany.

## 5. Conclusions

The present methodological description of the implementation of the AMBORA Center in clinical routine (1) adds knowledge on how to plan, structure, and perform a multicenter type III hybrid effectiveness–implementation trial using RE-AIM, (2) reports the conduction and preliminary results of semi-structured stakeholder interviews based on CFIR, and (3) proposes multilevel implementation strategies. Based on our findings, semi-structured stakeholder interviews are a valuable tool to identify barriers and facilitators at an early stage of the implementation process. Further research regarding the impact of an intensified clinical pharmacological/pharmaceutical care program on medication safety with oral antitumor therapy in real-world settings and the feasibility in daily clinical routine is required to derive recommendations for the dissemination to other sites (e.g., a best practice model).

## Data Availability

Not applicable.

## Supplementary Materials

The following supporting information can be downloaded at: https://www.mdpi.com/article/10.3390/healthcare11111640/s1, Figure S1: English translation of the interview guide, including all proposed questions in stakeholder interview #1 (original version in German), Figure S2: Detailed report of this type III hybrid trial according to the Standards for Reporting Implementation Studies (StaRI) guidelines [23]. Abbreviations: n.a. = not applicable, w.b.r. = will be reported after data completion.
