# Supplementary material for: Optimizing Medication Safety with Oral Antitumor Therapy: A Methodological Approach for the Real-World Implementation of the AMBORA Competence and Consultation Center
Source: Healthcare (Basel). 2023 Jun 3;11(11):1640. doi: 10.3390/healthcare11111640 (PMC10252727; doi:10.3390/healthcare11111640)
Supplement: Supplementary file 1 [file healthcare-11-01640-s001.zip › healthcare-2405602-supplementary.pdf]

Cuba et al. Optimizing Medication Safety with Oral Antitumor Therapy: A Methodological Approach for the Real-World Implementation of the AMBORA Competence and Consultation Center

Supplementary Materials

Detailed methodological approach for semi-structured stakeholder-interviews

Figure 4a presents the systematic multilevel methodological approach for developing semi-structured interview questionnaires based on the Consolidated Framework for Implementation Research (CFIR) [31]. First of all, two team members independently assessed all CFIR (sub-)constructs and preliminarily classified them into whether they are to be considered as relevant (n = 19, team member A and n = 12, team member B). Secondly, the selected (sub-)constructs were compared with the validated German version of CFIR [32] in order to avoid misconceptions due to linguistic barriers. This was followed by the reconciliation with an external, systematic review [33] displaying the most commonly used CFIR (sub-)constructs. After internal agreement, all CFIR (sub-)constructs that have been used in at least 7 out of the 15 reviewed studies are considered to be included in our semi-structured stakeholder interview questions (n = 14). Following aggregation and joint evaluation of all CFIR (sub-)constructs selected by the two team members and the systematic review, a total of 16 CFIR (sub-)constructs are to be considered as relevant for the development of the interview questions. Figure 4b displays the finally selected CFIR (sub-)constructs for stakeholder interview #1 and #2. Two team members formulated the interview questions independently with emphasis on clear and unambiguous phrasing. Following thorough discussion and refinement of all proposed questions, all five team members of the project group finally approved the interview guide. The English version of the questionnaire used as our final interview guide for stakeholder interviews #1 is displayed below.

Figure S1. English translation of the interview guide including all proposed questions in stakeholder interview #1 (original version in German).

FRIEDRICH-ALEXANDER UNIVERSITÄT ERLANGEN-NÜRNBERG MEDIZINISCHE FAKULTÄT — CCC Comprehensive Cancer Center Erlangen-EMN Europäische Metropolregion Nürnberg — Universitätsklinikum Erlangen

Stakeholder Interview #1 - Questionnaire

Aim:

We constantly evaluate the implementation process of the **AMBORA Competence and Consultation Center** (AMBORA Center; funded by the German Cancer Aid) in order to ensure a successful integration into clinical practice. For this purpose, we conduct semi-structured stakeholder interviews (e.g., with clinic directors, leading physicians, or resident practice owners) in all participating clinics and practices. These interviews will allow us to identify barriers and facilitators of the implementation process. The interview questions are based on the Consolidated Framework for Implementation Research (CFIR) [Damschroder et al., Implement Sci 2009].

1. Do you think that this implementation project is based on good scientific evidence with sufficiently high quality?

(Please mark where applicable.)

Yes, definitely	Yes, partly	Maybe	Rather not	Not at all
1	2	3	4	5

2. How would you grade the evidence-based clinical effects evaluated in the AMBORA trial [Dürr et al, JCO 2021] concerning their importance for the implementation in your clinic or practice?

(Please mark where applicable.) OAT = oral antitumor therapeutics.

	**Very important**	**Important**	**Partly important**	**Hardly important**	**Not important**
Reduction of OAT-related adverse events	1	2	3	4	5
Reduction of medication errors	1	2	3	4	5
Reduction of hospitalizations rates	1	2	3	4	5
Improvement of patients' quality of life	1	2	3	4	5
Improvement of patients' knowledge about the OAT	1	2	3	4	5
Improvement of patients' OAT treatment satisfaction	1	2	3	4	5
Reduction of OAT discontinuations	1	2	3	4	5
Others:	1	2	3	4	5
Others:	1	2	3	4	5

3. Do you consider the services of the AMBORA Center as a meaningful addendum in routine medical care for patients' health?

(Please mark where applicable.)

Yes, definitely	Yes, partly	Maybe	Rather not	Not at all
1	2	3	4	5

4. How would you grade the patient information leaflets concerning their quality and design?

(Please mark where applicable.)

Very good	Good	Satisfactory	Sufficient	Inadequate
1	2	3	4	5

5. How would you grade the services of the AMBORA Center concerning their usefulness in your daily clinical practice?

(Please mark where applicable.)

	**Very useful**	**Useful**	**Partly useful**	**Hardly useful**	**Not useful**
Patient consultation sessions	1	2	3	4	5
Medication reconciliations	1	2	3	4	5
Information leaflets	1	2	3	4	5
Quality improvement in patient care	1	2	3	4	5
Counseling service for healthcare professionals	1	2	3	4	5
Reduced time requirement for physicians	1	2	3	4	5
Reputation enhancement	1	2	3	4	5
Others:	1	2	3	4	5
Others:	1	2	3	4	5

6. Do you consider the implementation of the AMBORA Center into routine care of your clinic or practice as organizationally feasible?

(Please mark where applicable.)

Yes, definitely	Yes, partly	Maybe	Rather not	Not at all
1	2	3	4	5

7. What kind of organizational barriers (e.g., concerning seamless communication or collaboration with the AMBORA-team) do you anticipate in your clinic or practice?

(Please mark where applicable.)

☐ Unavailable rooms for patient consultation sessions	☐ Poor motivation for patient referral
☐ Time or personnel resources for patient referral	☐ Competing ongoing trials
☐ Limited access to patient records	☐ Others:

8. What conditions does our service need to fulfill in order to permanently integrate it into routine care of your clinic or practice?

(Please mark where applicable.)

- ☐ Certain percentage of cancer patients, who are supported by the AMBORA-consultation team in your clinic or practice (at least _____ %)
- ☐ Reimbursement of the service by health insurances (clarification of financing)
- ☐ Automated, electronic request of consultations to avoid active contacting of the AMBORA- team (e.g., using MEONA®)
- ☐ Very good feedback from patients
- ☐ Very good feedback from physicians / nurses
- ☐ Dissemination of similar centers to others sites
- ☐ Others:
- ☐ Others:

9. Interview notes / other information.

Figure S2. Detailed report of this type III hybrid trial according to the Standards for Reporting Implementation Studies (StaRI) guidelines [23].

Standards for Reporting Implementation Studies: the StaRI checklist for completion

The StaRI standard should be referenced as: Pinnock H, Barwick M, Carpenter C, Eldridge S, Grandes G, Griffiths CJ, Rycroft-Malone J, Meissner P, Murray E, Patel A, Sheikh A, Taylor SJC for the StaRI Group. Standards for Reporting Implementation Studies (StaRI) statement. BMJ 2017;356:i6795

The detailed Explanation and Elaboration document, which provides the rationale and exemplar text for all these items is: Pinnock H, Barwick M, Carpenter C, Eldridge S, Grandes G, Griffiths C, Rycroft-Malone J, Meissner P, Murray E, Patel A, Sheikh A, Taylor S, for the StaRI group. Standards for Reporting Implementation Studies (StaRI). Explanation and Elaboration document. BMJ Open 2017 2017;7:e013318

Notes: A key concept of the StaRI standards is the dual strands of describing, on the one hand, the implementation strategy and, on the other, the clinical, healthcare, or public health intervention that is being implemented. These strands are represented as two columns in the checklist. The StaRI standards refer to the broad range of study designs employed in implementation science. Authors should refer to other reporting standards for advice on reporting specific methodological features. Conversely, whilst all items are worthy of consideration, not all items will be applicable to, or feasible within every study.

StaRI

The primary focus of implementation science is the implementation strategy (column 1) and the expectation is that this will always be completed.

The evidence about the impact of the intervention on the targeted population should always be considered (column 2) and either health outcomes reported or robust evidence cited to support a known beneficial effect of the intervention on the health of individuals or populations.

Checklist item		Reported on page #	Implementation Strategy	Reported on page #	Intervention
			"Implementation strategy" refers to how the intervention was implemented		"Intervention" refers to the healthcare or public health intervention that is being implemented.
**Title and abstract**					
Title	1	1	Identification as an implementation study, and description of the methodology in the title and/or keywords		
Abstract	2	1	Identification as an implementation study, including a description of the implementation strategy to be tested, the evidence-based intervention being implemented, and defining the key implementation and health outcomes.		
**Introduction**					
Introduction	3	1-2	Description of the problem, challenge or deficiency in healthcare or public health that the intervention being implemented aims to address.		
Rationale	4	1-2	The scientific background and rationale for the implementation strategy (including any underpinning theory/framework/model, how it is expected to achieve its effects and any pilot work).	1-2	The scientific background and rationale for the intervention being implemented (including evidence about its effectiveness and how it is expected to achieve its effects).
Aims and objectives	5	2 (+ Fig 2)	The aims of the study, differentiating between implementation objectives and any intervention objectives.		
**Methods: description**					
Design	6	2-3	The design and key features of the evaluation, (cross referencing to any appropriate methodology reporting standards) and any changes to study protocol, with reasons		
Context	7	2 (+ 11)	The context in which the intervention was implemented. (Consider social, economic, policy, healthcare, organisational barriers and facilitators that might influence implementation elsewhere).		
Targeted 'sites'	8	2-3 (+ 8)	The characteristics of the targeted 'site(s)' (e.g locations/personnel/resources etc.) for implementation and any eligibility criteria.	3, 5	The population targeted by the intervention and any eligibility criteria.
Description	9	5-7 (+ Fig 3)	A description of the implementation strategy	2-5 (+ Fig 1)	A description of the intervention
Sub-groups	10	n.a.	Any sub-groups recruited for additional research tasks, and/or nested studies are described		

**Methods: evaluation**					
Outcomes	11	3-8 (+ Fig 2)	Defined pre-specified primary and other outcome(s) of the implementation strategy, and how they were assessed. Document any pre-determined targets	3-8 (+ Fig 2)	Defined pre-specified primary and other outcome(s) of the intervention (if assessed), and how they were assessed. Document any pre-determined targets
Process evaluation	12	3-8	Process evaluation objectives and outcomes related to the mechanism by which the strategy is expected to work		
Economic evaluation	13	3-8	Methods for resource use, costs, economic outcomes and analysis for the implementation strategy	3-8	Methods for resource use, costs, economic outcomes and analysis for the intervention
Sample size	14	n.a.	Rationale for sample sizes (including sample size calculations, budgetary constraints, practical considerations, data saturation, as appropriate)		
Analysis	15	3-8	Methods of analysis (with reasons for that choice)		
Sub-group analyses	16	n.a.	Any a priori sub-group analyses (e.g. between different sites in a multicentre study, different clinical or demographic populations), and sub-groups recruited to specific nested research tasks		
**Results**					
Characteris-tics	17	w.b.r.	Proportion recruited and characteristics of the recipient population for the implementation strategy	8	Proportion recruited and characteristics (if appropriate) of the recipient population for the intervention
Outcomes	18	8-9 (+ Fig 5)	Primary and other outcome(s) of the implementation strategy	w.b.r.	Primary and other outcome(s) of the Intervention (if assessed)
Process outcomes	19	w.b.r.	Process data related to the implementation strategy mapped to the mechanism by which the strategy is expected to work		
Economic evaluation	20	w.b.r.	Resource use, costs, economic outcomes and analysis for the implementation strategy	w.b.r.	Resource use, costs, economic outcomes and analysis for the intervention
Sub-group analyses	21	n.a.	Representativeness and outcomes of subgroups including those recruited to specific research tasks		
Fidelity/ adaptation	22	w.b.r.	Fidelity to implementation strategy as planned and adaptation to suit context and preferences	w.b.r.	Fidelity to delivering the core components of intervention (where measured)
Contextual changes	23	n.a.	Contextual changes (if any) which may have affected outcomes		
Harms	24	w.b.r.	All important harms or unintended effects in each group		
**Discussion**					
Structured discussion	25	10-12	Summary of findings, strengths and limitations, comparisons with other studies, conclusions and implications		
Implications	26	10-12	Discussion of policy, practice and/or research implications of the implementation strategy (specifically including scalability)	10-12	Discussion of policy, practice and/or research implications of the intervention (specifically including sustainability)
**General**					
Statements	27	2-3	Include statement(s) on regulatory approvals (including, as appropriate, ethical approval, confidential use of routine data, governance approval), trial/study registration (availability of protocol), funding and conflicts of interest		

Abbreviations: n.a. = not applicable, w.b.r. = will be reported after data completion.
